# VALIDATION OF DEMENTIA CARE-RELATED SCALES AMONG INFORMAL CAREGIVERS OF LATINOS WITH DEMENTIA OR MCI

**DOI:** 10.1093/geroni/igae098.3765

**Published:** 2024-12-31

**Authors:** Jaime Perales-Puchalt, Monica Fracachan Cabrera, Christina Baker, Mariana Ramirez Mantilla, Jeffrey Burns

**Affiliations:** University of Kansas Medical Center, Fairway, Kansas, United States; University of Kansas Medical Center, Kansas City, Kansas, United States; University of Kansas Medical Center, Kansas City, Kansas, United States; University of Kansas Medical Center, Kansas City, Kansas, United States; University of Kansas Medical Center, Fairway, Kansas, United States

## Abstract

We aimed to test the psychometric properties of several dementia care-related scales among Latinos. We leveraged secondary baseline data from a one-arm mHealth trial on dementia caregiver support. We included 100 responses for caregiver-focused scales and 88 responses for care recipient-focused scales. Scales included the Neuropsychiatric Inventory Questionnaire Severity and Distress scales, six-item Zarit Burden Inventory, Ten-item Center for Epidemiologic Studies Depression Scale, Geriatric Depression Inventory, Quality of Life in Alzheimer’s Disease, and Single-item Satisfaction With Life Scale. We calculated concurrent validity using Pearson and Spearman correlations and expected correlations amongst all variables in line with the Stress Process Framework. We calculated internal consistency reliability using Cronbach’s alpha. All concurrent validity correlations followed the expected directionality, with 19/21 inter-scale correlations in the total sample reaching statistical significance (p< 0.05), and 17/21 reaching at least a low correlation (0.3). Cronbach’s alpha ranged from 0.832 and 0.879 in all scales in the total sample. Findings suggest that these English and Spanish caregiver-administered scales have good psychometric properties.

